# Treatment of pediatric spondylolysis and spondylolisthesis

**DOI:** 10.2340/17453674.2024.42450

**Published:** 2025-01-13

**Authors:** Ilkka HELENIUS, Ella VIRKKI, Taavi TOOMELA, Daniel STUDER, Martin GEHRCHEN, Matti AHONEN

**Affiliations:** 1 Department of Orthopaedics and Traumatology, University of Helsinki and Helsinki University Hospital, Helsinki, Finland; 2 Department of Paediatric Surgery, Orthopaedics and Traumatology, University of Turku and Turku University Hospital, Finland; 3 East Tallinn Central Hospital, Tallinn, Estonia; 4 Department of Orthopaedic Surgery, University Children’s Hospital Basel, Basel, Switzerland; 5 Department of Orthopaedic Surgery, Rigshospitalet and University of Copenhagen, Copenhagen, Denmark; 6 Helsinki New Children’s Hospital, Helsinki University Hospital, Helsinki, Finland

## Abstract

Spondylolysis is defined as a defect or elongation in the pars interarticularis of the lumbar spine, either unilateral or bilateral. Growing children with bilateral spondylolysis may develop spondylolisthesis, i.e., forward slipping of the affected vertebra. The etiology of spondylolysis is regarded as a stress fracture due to repetitive loading associated with a genetic predisposition. Lumbar magnetic resonance imaging (MRI) shows an increased signal intensity before an actual fracture line develops. In low grade spondylolisthesis, two-thirds of children with acute pediatric spondylolysis will undergo bony union with early activity restriction. Health-related quality of life is improved in patients achieving bony union as compared with patients having non-union, of which one-fourth will additionally develop spondylolisthesis. In patients with high-grade spondylolisthesis, defined as a more than 50% forward slippage of the affected vertebra, spinal fusion is recommended to prevent further progression.

## Take-home messages

- Early identification of lumbar stress fracture (spondylolysis) and activity restriction allows healing of the stress fracture in two-thirds of affected children. Brace treatment is not necessary for bony union.
- Union of the stress fracture predicts improved health-related quality of life in physically active adolescents.
- There is a 25% risk of low-grade spondylolisthesis in children with non-union spondylolysis.
- Most adolescents with low-grade spondylolisthesis can be treated non-surgically.
- High-grade spondylolisthesis (> 50% slip) is an indication for instrumented spinal fusion to prevent further progression and to improve function and outcome.
- Selection of fusion levels (L5–S1 vs L4–S1) depends on the sagittal alignment.

The aim of this educational review is to update the reader on recent findings on the classification, non-surgical, and surgical management of pediatric spondylolysis and on spondylolisthesis.

Lumbar spondylolysis refers to a defect or elongation of the pars interarticularis and can be uni- or bilateral. Natural history studies suggest that 4.4% of 6-year-old children and 6% of young adults have a radiographic spondylolysis [1]. It has been estimated that low back pain in physically active teenagers is caused by spondylolysis in 50% [2]. The etiology of spondylolysis has been regarded as a stress fracture in children with genetic predisposition as spondylolysis was not observed in 500 newborns [1,3]. Additionally, spondylolysis does not develop in neurologically affected children who cannot stand up [4] and the prevalence of spondylolysis is higher in teenage athletes [5,6].

**Figure 1:**
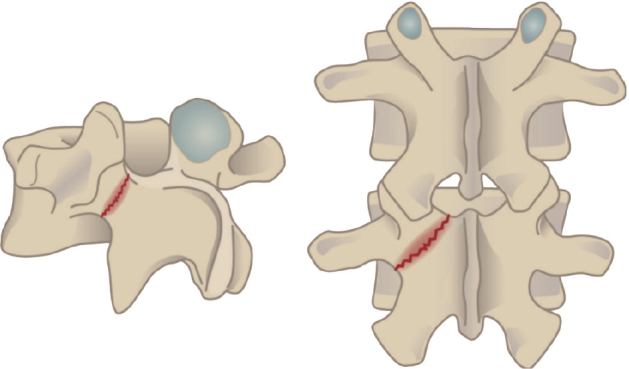
Stress fracture of the pars interarticularis.

Spondylolysis starts on the ventral surface of the pars interarticularis due to compression of the upper facet joint of the inferior vertebra (Figure 1). Children who perform repeated lumbar extension activities combined with rotational movements such as in gymnastics, ballet, and figure skating have a high risk of developing stress fracture. Lumbar lordosis develops individually based on pelvic geometry (pelvic incidence). A larger pelvic incidence results in larger lumbar lordosis and increased risk of spondylolysis [7]. Kriz et al. [8] evaluated 902 young athletes with painful pars interarticularis and pedicle stress injuries using lumbar MRI. Of these, 65% had L5, 24% L4, 8.4% L3, 1.4% L2, and none L1 stress injuries. Pars or pedicle stress fracture was observed in 59% of all patients, and only 7.1% had a fracture at or above the L3 level.

Growing children with bilateral spondylolysis may develop spondylolisthesis, i.e., forward slippage of the affected vertebra. Spondylolisthesis may also develop without a defect in the pars interarticularis due to facet joint dysplasia, which may be accompanied by elongation of the pars interarticularis or spina bifida occulta and is called dysplastic spondylolisthesis [9,10]. Development of both dysplastic and isthmic spondylolisthesis seem to be associated with genetic predisposition [11].

## Classification

**Figure 2:**
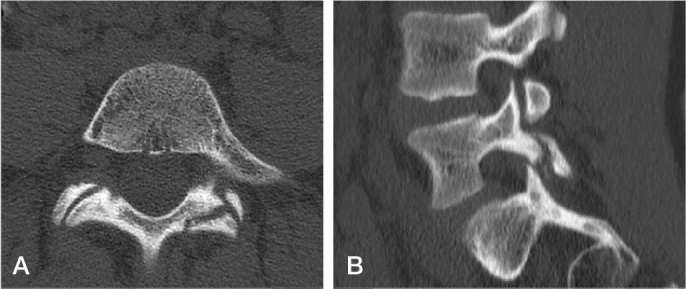
Progressive unilateral spondylolysis in the pars interarticularis of the L5. (A) axial CT, (B) sagittal reformatting.

Stress fracture can be classified as stress osteopathy (signal intensity change without a fracture line on MR images), early (hairline fracture line on CT), progressive (gap in the fracture line), and terminal (rounded edges of the fracture line, bony sclerosis) [12,13] (Figure 2).

**Figure 3:**
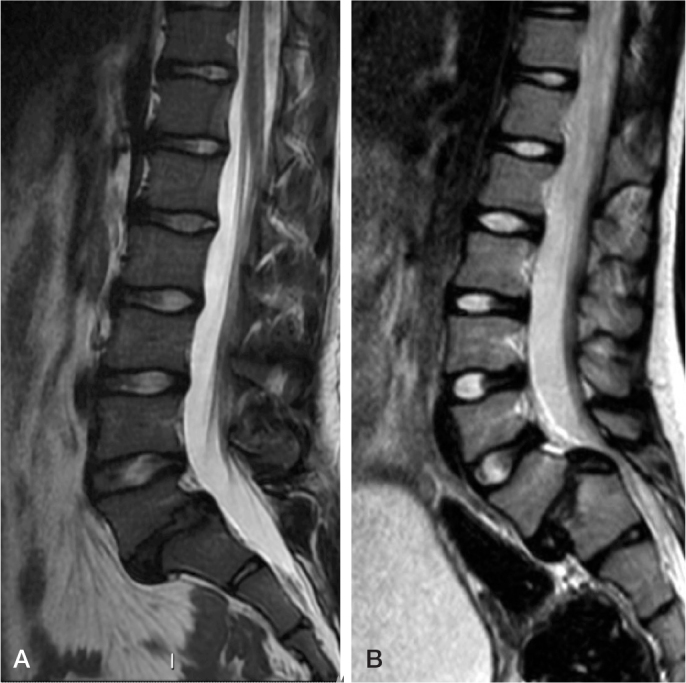
(A) Isthmic and (B) dysplastic spondylolisthesis in T2 weighted MR images. Central spinal canal remains wide in the isthmic lesion, while in the dysplastic lesion it has narrowed. The sacrum has developed a ridge necessitating sacral osteotomy (ridge resection) to facilitate reduction of the forward slip and bony healing.

Wiltse et al. [14] classified spondylolysis and spondylolisthesis according to etiology into dysplastic (type I), isthmic (stress fracture, type II), degenerative (type III), iatrogenic (type IV), and pathologic (type V) [14,15]. Children mainly have type I and type II lesions. Patients with dysplastic spondylolisthesis (type I) typically present with spina bifida, congenital malformation of facet joints, trapezoidal L5, and rounding of the sacral endplate. Type II can further be classified as stress fracture of pars interarticularis, elongated pars interarticularis, and traumatic fracture. Patients with dysplastic high-grade spondylolisthesis have a risk of developing neurologic deficit as the intact posterior elements may produce compression on the L5 nerve roots and cauda equina [9,10]. In isthmic spondylolisthesis the central spinal canal remains wide, and L5 nerve root compression will become evident only after disc degeneration (Figure 3).

Meyerding classified spondylolisthesis according to the amount of forward slippage of the vertebra [16]. Grade I spondylolisthesis is less than 25% slippage, grade II as slippage of 25–49%, grade III 50–74% slippage, and grade IV 75–99% slippage. Spondyloptosis, meaning full forward slippage (100%) of the lumbar vertebra in front of the sacrum, represents grade V. Slippage of less than 50% is typically grouped into low-grade spondylolisthesis and slippage of 50% or more as high-grade spondylolisthesis. The Spinal Deformity Study Group classifies high-grade spondylolisthesis further according to compensatory mechanisms to retain an upright posture: balanced pelvis (no pelvic retroversion), unbalanced pelvis (pelvic retroversion with increased pelvic tilt and reduced sacral slope), and unbalanced pelvis and unbalanced spine (global sagittal spinal balance in front of the femoral heads) [17].

## Spondylolysis and low-grade spondylolisthesis

**Figure 4:**
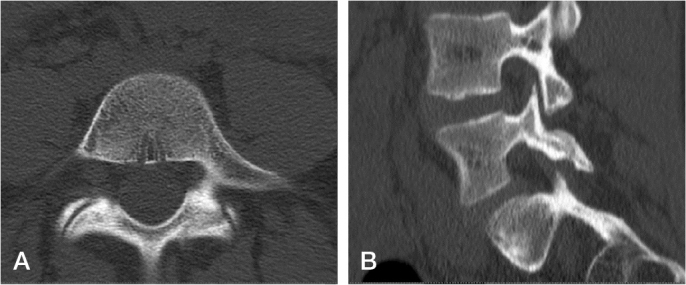
Radiographic healing of the progressive unilateral spondylolysis on CT after 4 months’ activity restriction. (A) Axial and (B) sagittal reformatting demonstrate bony bridging of the stress fracture, although trabeculation is not complete.

**Figure 5:**
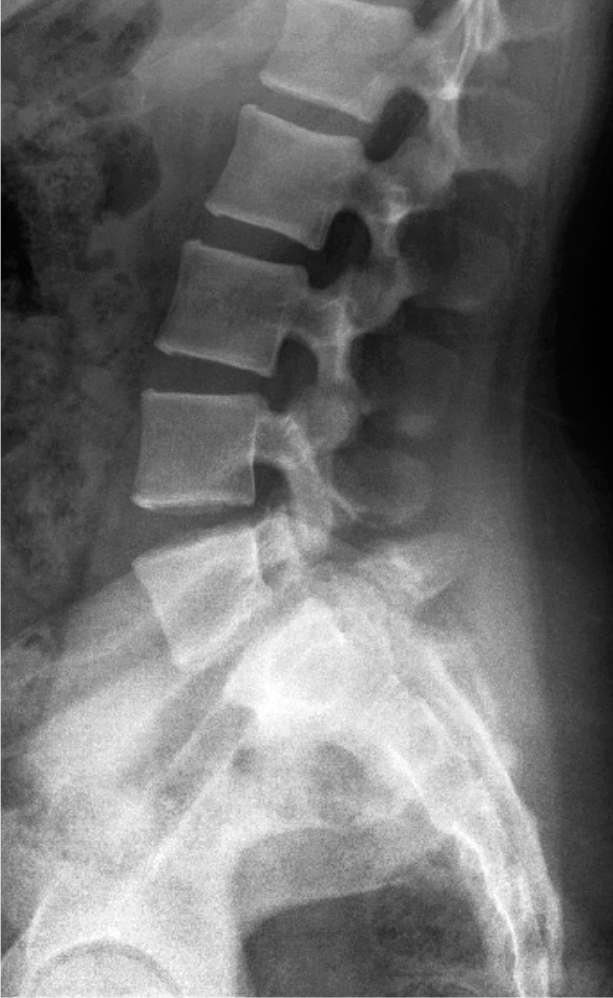
Low-grade spondylolisthesis at 2-year follow-up radiograph after non-union of bilateral L5 spondylolysis.

Acute pediatric spondylolysis has a chance of bony union depending on the stage of the defect (Figure 4) [12,18]. Stress osteopathy without a fracture line represents a self-limiting condition with activity restrictions but tends to recur. Sakai et al. [13] followed patients with acute spondylolysis using monthly MRI. Healing of the defect was noted at a mean of 14 weeks’ follow-up. Sairyo et al. [19] noted that a high signal change in the adjacent pedicle on a T2-weighted MRI significantly increased the possibility of bony union in early-stage lesions. MRI is a valuable tool for detecting bone edema, and its diagnostic accuracy in identifying pars fractures has been improved using the T1 VIBE sequence, eliminating the need for a CT scan in detection of pars fractures [20]. In a recent prospective study, early lesions with partial (early) or complete fracture line (progressive) healed on a CT scan in 67% of the patients with 4 months’ activity restriction [18]. The eligible criteria of this study included a high signal intensity on MRI. Walking and cycling were allowed during the activity restriction period along with isometric trunk muscle training. Immobilization using a rigid thoracolumbosacral orthosis did not improve union rates [18]. Patients achieving bony union had improved health-related quality of life (Scoliosis Research Society–24 outcome questionnaire total score) as compared with patients having non-union [21]. 25% of patients with bilateral spondylolysis progressed to low-grade spondylolisthesis during 2-year follow-up (Figure 5). Bony union is more common in patients with a unilateral defect or defect in L4, whereas trapezoidal L5, rounding of the sacral dome, and > 5% spondylolisthesis increase the non-union risk [12]. Patients with terminal defects (rounded edges or sclerosis of the fracture line) will not heal with conservative treatment [12]. The clinical implications of these studies suggest that early identification of an acute pediatric spondylolysis and activity restriction for 4 months is beneficial, as obtaining bony union of the spondylolysis will improve the functional outcome [21].

**Figure 6:**
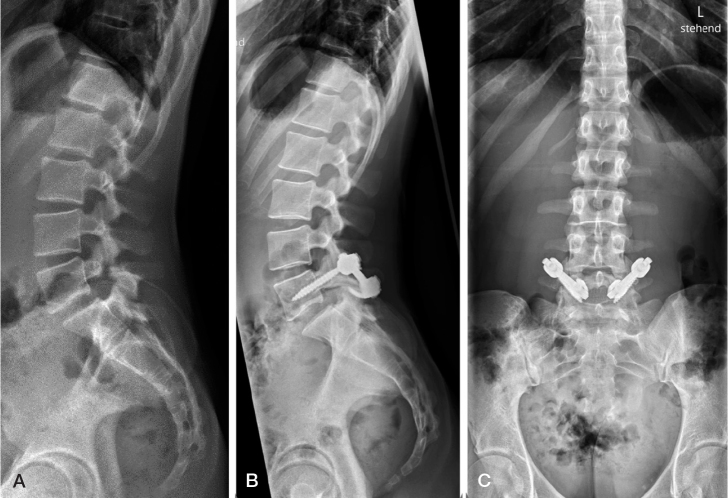
(A) Bilateral L5 spondylolysis. (B, C) Revision of the pseudoarthrosis with bilateral pedicle screw and hook instrumentation.

Most patients with spondylolysis do well even after non-union of the spondylolysis but approximately one-fifth will develop low back pain [22]. Patients with unilateral defects are at risk of developing spondylolysis on the contralateral side. Patients with persisting symptoms of bilateral spondylolysis can be treated operatively either by performing a resection of the pseudarthrosis in combination with a direct repair of the pars defect or by performing a posterolateral spinal fusion of the affected vertebra and the vertebra below (Figure 6). Studies comparing these 2 techniques showed better long-term health-related quality of life values for patients undergoing posterolateral fusion [23].

The prognosis of low-grade spondylolisthesis in children is generally good. Only a few of these patients develop persisting low back or radicular pain over time. Progression of the slip of ≥ 10% was observed in 3 out of 72 children over a 13-year follow-up period [24]. Routine radiographic follow-up is recommended in children with significant remaining growth. Our practice is to follow these patients yearly over the growth spurt and have additional follow-ups if symptoms occur. Non-surgical treatment using activity restriction with lumbar segmental stabilization exercises (“core stability”) is usually effective in alleviating symptoms [25]. Therefore, surgical management remains the second line of choice in these patients, although lower rates of low back pain have been reported for surgically treated patients in mid- and long-term follow-up studies [26,27].

## High-grade spondylolisthesis

High-grade spondylolisthesis (> 50% slip) tends to progress in growing children with non-surgical management. 6 of 11 non-surgically treated adolescents with high-grade spondylolisthesis (Meyerding grade III or IV) progressed to spondyloptosis during a mean of 18 years’ follow-up [28]. Additionally, 5 of these patients had neurological symptoms at the end of follow-up. In contrast, 20 of the 21 surgically treated patients (posterior in situ fusion without decompression) had minimum or no symptoms at a mean of 24-year follow-up for high-grade spondylolisthesis. In patients with high pelvic incidence and sacral slope, shear forces may play a rolein progression [29]. Patients with high-grade spondylolisthesis may show increasing slip after posterolateral fusion as the fusion mass may bend [24,30]. It should be noted, however, that the true radiographic progression of high-grade spondylolisthesis may be difficult to measure due to radiographic measurement error [31]. A long-term study has suggested an improved health-related quality of life in patients with high-grade spondylolisthesis receiving circumferential fusion (interbody and posterolateral) as compared with posterior or anterior only non-instrumented spinal fusion [32]. Non-instrumented spinal fusion carries a 20% risk of non-union during follow-up, especially in growing children [33], and therefore most authors prefer instrumented spinal fusion. Cauda equina syndrome is a potential complication for both in situ fusion [9] and after instrumented reduction [34,35].

Different opinions exist regarding the role of instrumented reduction of high-grade spondylolisthesis [22]. In situ fusion with or without nerve root decompression has been stated to reduce risk of L5 nerve root deficits when compared with instrumented reduction, which requires wide neural element decompression (L5 nerve roots and cauda equina) and increases transiently L5 nerve root tension [36]. Poussa et al. [37] compared the long-term outcomes of 11 patients undergoing instrumented reduction and circumferential spinal fusion with 11 patients undergoing non-instrumented circumferential spinal fusion. The health-related quality of life outcomes (Oswestry Disability Index and Scoliosis Research Society–24 outcome questionnaire) were better in the non-reduction group at a mean of 14.8-year follow-up [37]. In an evidence-based analysis Longo et al. [38] observed higher union rate in patients undergoing instrumented reduction as compared with in situ fusion with no increased risk of motor deficits.

Different opinions exist also on the length of spinal fusion in high-grade spondylolisthesis (L4–S1 vs L5–S1). Most authors have advocated longer fusion from L4–S1 to prevent adjacent-segment instability [39], while this will sacrifice the L4–L5 disc [40]. Recently, Jiao et al. [41] compared the radiographic outcomes of L5–S1 and L4–S1 instrumented spinal fusion for pediatric high-grade spondylolisthesis. They observed adjacent segment instability (> 3 mm spondylolisthesis or posterior opening of more than 5° between L4 and L5) in 13 of 53 patients having short vs 0 of 15 patients with longer fusion.

**Figure 7:**
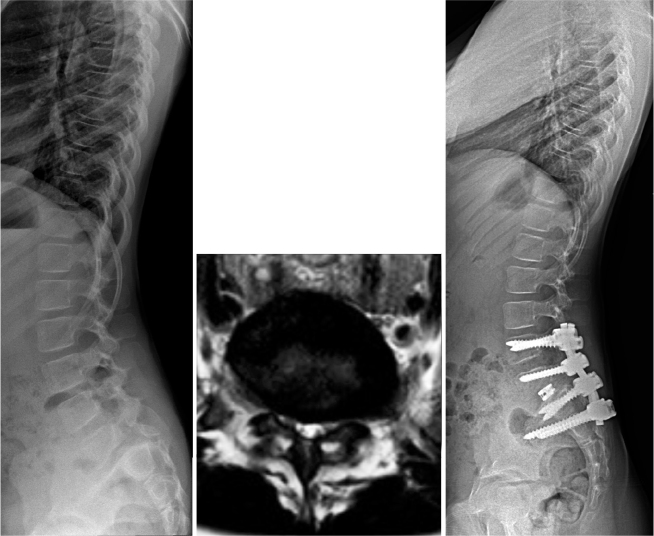
High-grade spondylolisthesis in a 9-year-old girl. (A) Standing lateral radiograph shows pelvic retroversion (increased pelvic tilt) and lower thoracic lordosis as a sign of compensatory mechanisms for sagittal imbalance. (B) Axial T2 weighted MR image shows central canal stenosis typical of dysplastic high-grade spondylolisthesis. (C) Instrumented spinal fusion from L4 to S1 was supplemented by iliac screws to prevent bending of sacrum between S1 and S2 disc space. Reduction was facilitated by sacral dome osteotomy and interbody fusion was carried out to increase the possibilities for circumferential spinal fusion.

The clinical practice of the authors is to perform an instrumented reduction, nerve root decompression, and monosegmental (L5–S1) fusion for patients without sagittal balance compensatory mechanisms and longer fusion (L4–S1) in patients with unbalanced pelvis or with global spinal balance in front of the femoral heads (unbalanced spine) [42]. L4 could also be involved for securing correction, to prevent L5 pedicle screw pullout and promote spinal fusion. Sacral dome osteotomy may reduce the risk of L5 nerve root tension by shortening the spinal column and provides cancellous bony surface to facilitate interbody spinal fusion [43]. In children with open S1/S2 disc, bending of the sacrum can be prevented by supplementary iliac fixation (Figure 7). This approach is supported by a recent retrospective study on 61 patients with high-grade spondylolisthesis, where restoration of pelvic balance also improved the health-related quality of life outcomes [44].

**Figure 8:**
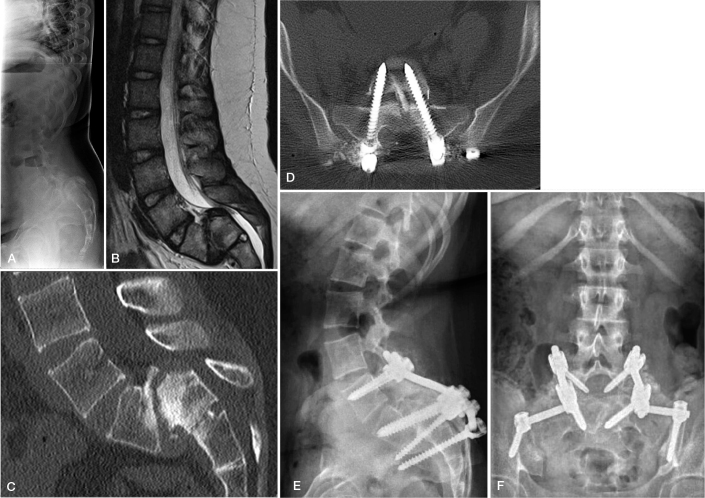
Spondyloptosis in a 12-year-old girl with bilateral L5 radicular symptoms. (A) Standing radiograph shows pelvic retroversion. (B) Sagittal T2 weighted MR image shows spondyloptosis with narrowing central spinal canal. (C) Sagittal reformatting of lumbar CT shows remodeling of the S1 endplate with residual apophyseal growth plate. (D) Axial CT image with trans-sacral screw fixation with a non-vascular fibular strut. (E) Standing lateral and (F) posteroanterior radiograph at 2-year follow-up shows bridging bone formation posterolaterally and reduced pelvic retroversion.

Spondyloptosis (L5 vertebral body in front of the sacrum) represents an even more difficult condition. Options include L5 vertebrectomy [45] or trans-sacral in situ fusion [46]. L5 vertebrectomy carries a 30% risk of transient or permanent L5 nerve root deficit. Most spinal surgeons including the authors prefer therefore to stabilize spondyloptosis using trans-sacral screws in situ instead of attempted vertebrectomy and reduction of L4 on top of the sacrum (Figure 8).

## Conclusions

Early identification of lumbar acute spondylolysis using MRI increases the likelihood of bony healing in children using activity restriction without immobilization. Bony healing improves the health-related quality of life and prevents the development of low-grade spondylolisthesis. Most children with low-grade spondylolisthesis have a favorable prognosis and only 4% are at risk of further slip progression. High-grade spondylolisthesis has a high risk of progression in growing children and should be surgically stabilized. Different opinions exist regarding the best surgical treatment option, but studies suggest that instrumented reduction may improve fusion rates [38].
